# Supplementary figures and images for: Differential clinical characteristics across traditional Chinese medicine (TCM) Syndromes in patients with sickle cell disease
Source: Front Pain Res (Lausanne). 2024 Jan 5;4:1233293. doi: 10.3389/fpain.2023.1233293 (PMC10796810; doi:10.3389/fpain.2023.1233293)

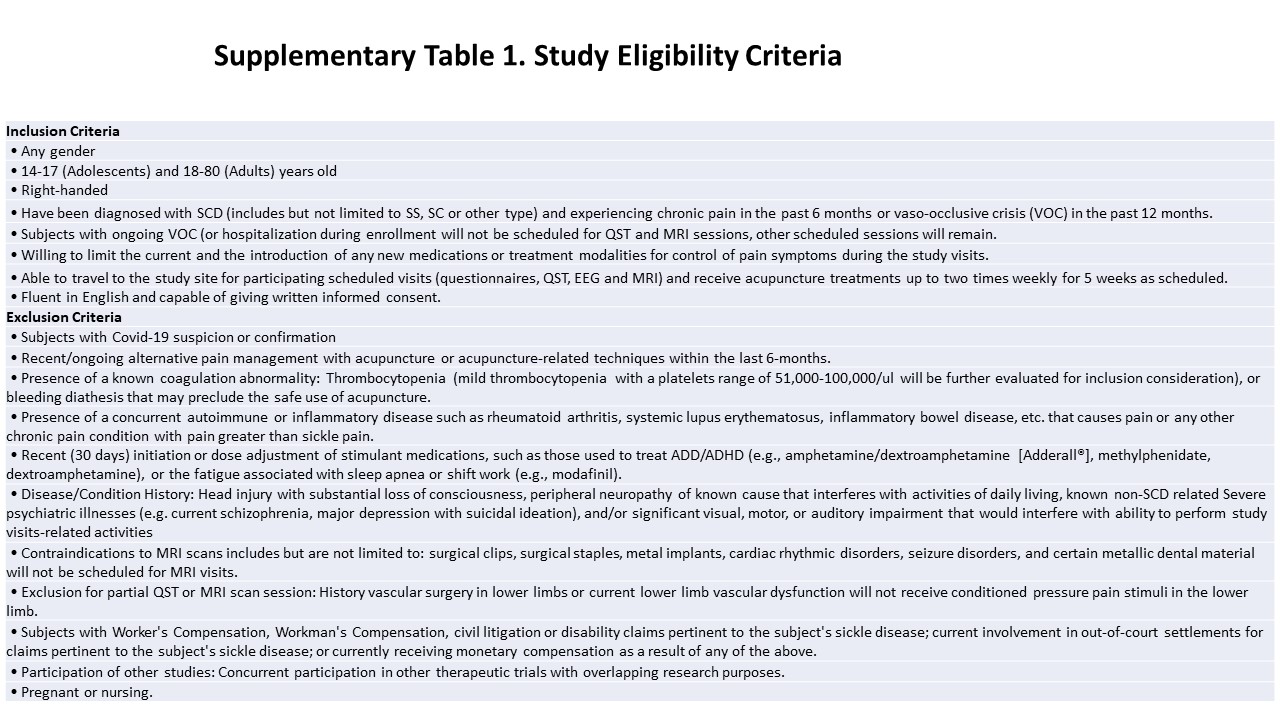

Supplement: Supplementary file 1 [file Image1.jpeg]

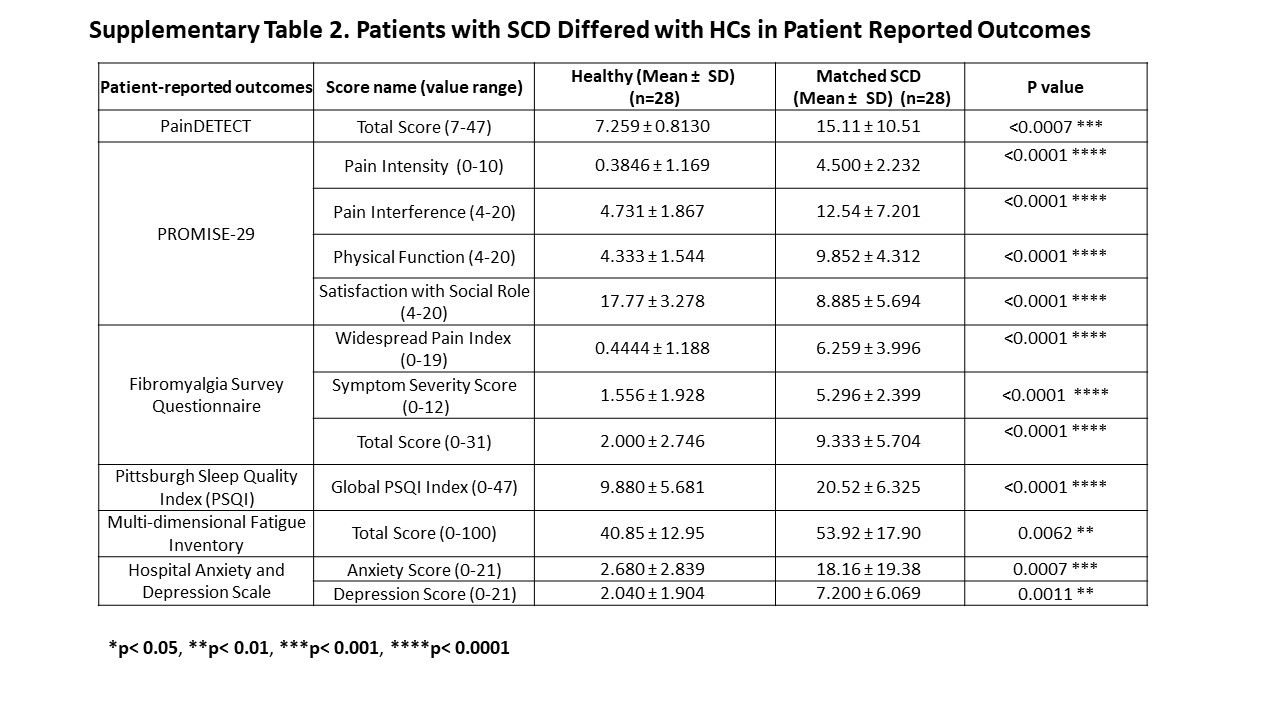

Supplement: Supplementary file 2 [file Image2.jpg]

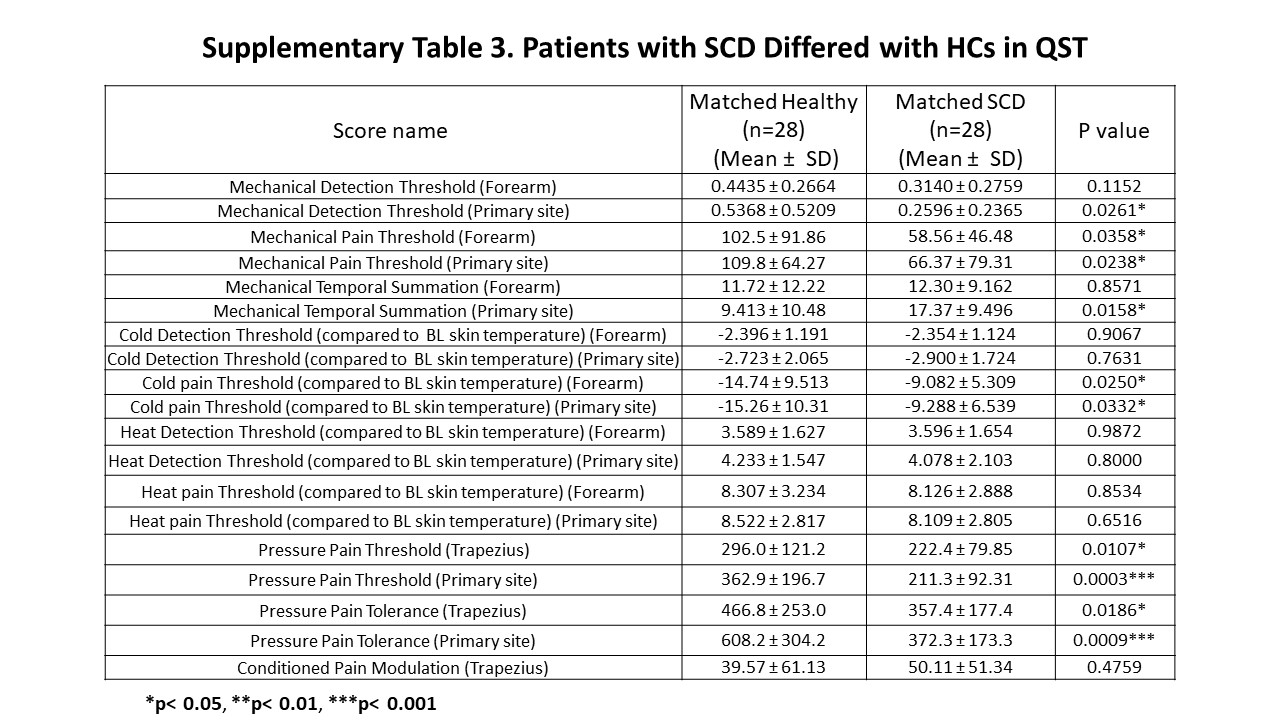

Supplement: Supplementary file 3 [file Image3.jpg]

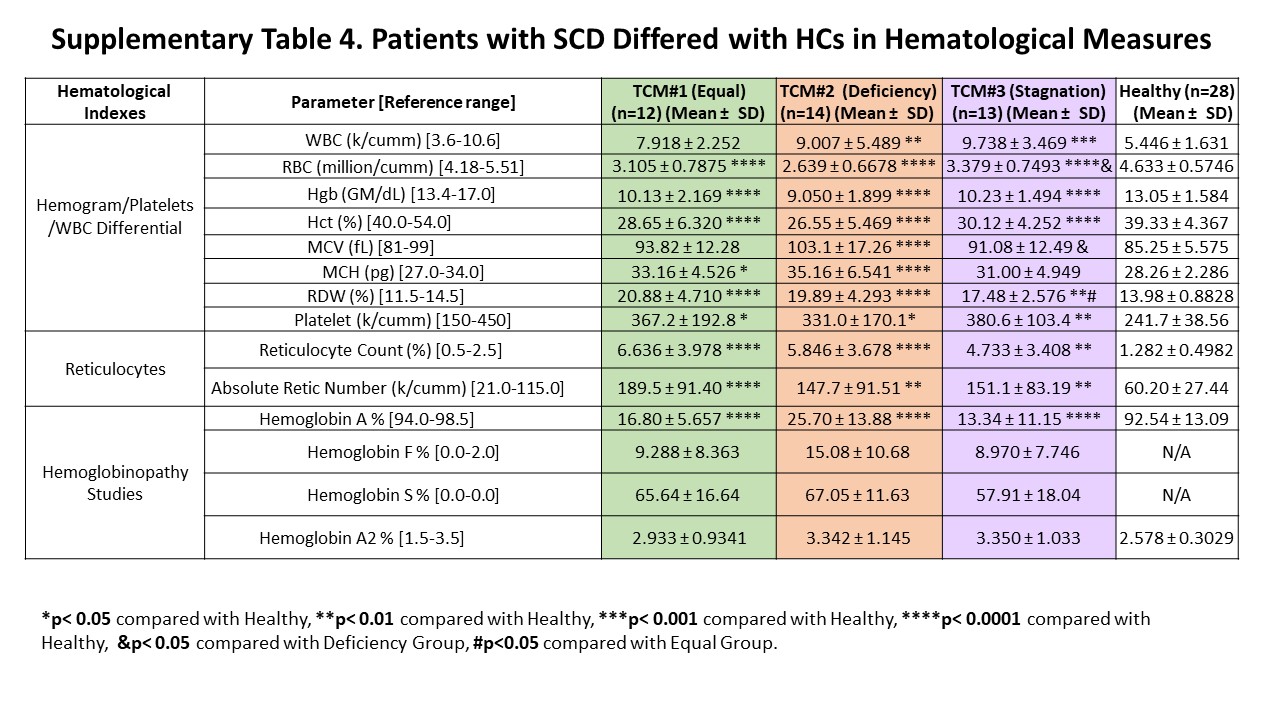

Supplement: Supplementary file 4 [file Image4.jpg]
